# Neural circuit mechanisms of post–traumatic epilepsy

**DOI:** 10.3389/fncel.2013.00089

**Published:** 2013-06-18

**Authors:** Robert F. Hunt, Jeffery A. Boychuk, Bret N. Smith

**Affiliations:** ^1^Epilepsy Research Laboratory, Department of Neurological Surgery, University of CaliforniaSan Francisco, CA, USA; ^2^Department of Physiology, College of Medicine, University of KentuckyLexington, KY, USA; ^3^Spinal Cord and Brain Injury Research Center, University of KentuckyLexington, KY, USA

**Keywords:** epilepsy, epileptogenesis, neuroinflammation, pilocarpine, seizures, synapse, traumatic brain injury

## Abstract

Traumatic brain injury (TBI) greatly increases the risk for a number of mental health problems and is one of the most common causes of medically intractable epilepsy in humans. Several models of TBI have been developed to investigate the relationship between trauma, seizures, and epilepsy-related changes in neural circuit function. These studies have shown that the brain initiates immediate neuronal and glial responses following an injury, usually leading to significant cell loss in areas of the injured brain. Over time, long-term changes in the organization of neural circuits, particularly in neocortex and hippocampus, lead to an imbalance between excitatory and inhibitory neurotransmission and increased risk for spontaneous seizures. These include alterations to inhibitory interneurons and formation of new, excessive recurrent excitatory synaptic connectivity. Here, we review *in vivo* models of TBI as well as key cellular mechanisms of synaptic reorganization associated with post-traumatic epilepsy (PTE). The potential role of inflammation and increased blood–brain barrier permeability in the pathophysiology of PTE is also discussed. A better understanding of mechanisms that promote the generation of epileptic activity versus those that promote compensatory brain repair and functional recovery should aid development of successful new therapies for PTE.

## INTRODUCTION

Post-traumatic epilepsy (PTE) is a common long-term consequence of traumatic brain injury (TBI), for which there are few effective therapies. More than one million people are presented for medical care each year in the United States after sustaining a head injury (Faul et al., 2010). While the incidence of epilepsy in the general population is about 1–2% (Hauser and Hesdorffer, 1990), the overall incidence of epilepsy is 7–39% after severe closed-head injury and as high as 57% after penetrating injury (Caveness et al., 1979; Salazar et al., 1985; Annegers et al., 1998; Asikainen et al., 1999; Herman, 2002; Englander et al., 2003), and approximately 20% of all acquired epilepsies are the result of TBI (Hauser et al., 1991). Injury severity is generally considered the major determining factor for developing a seizure disorder after brain trauma (Annegers et al., 1998; Herman, 2002; Englander et al., 2003). Focal brain damage and cortical contusions to the frontal, temporal, or parietal lobes are also important risk factor for human PTE (D’Alessandro et al., 1982; Englander et al., 2003). Several other risk factors have been identified, many of which are associated with focal lesions: dural penetration, depressed skull fracture, intraparenchymal hemorrhage, epidural and/or subdural hematomas, reduced brain volume, prolonged impaired consciousness, presence of injury-induced seizures, age (young children and the elderly), and gender (male; Salazar et al., 1985; Annegers et al., 1998; Asikainen et al., 1999; Englander et al., 2003; Frey, 2003). PTE occurs as temporal lobe epilepsy (TLE) in 35–62% of trauma patients (Diaz-Arrastia et al., 2000; Hudak et al., 2004).

Here, we review experimental animal models used in PTE research, including the neurophysiological and structural abnormalities believed to underlie the increased propensity of the injured brain to generate spontaneous seizures. Emphasis is placed on modifications of synaptic networks in the injured dentate gyrus that are associated with post-traumatic epileptogenesis. The hippocampus has long been recognized as an important structure in epilepsy (Lothman et al., 1991). The dentate gyrus is particularly susceptible to injury, often undergoes time-dependent structural reorganization, is a widely used model system for altered synaptic circuitry of cortical structures in epilepsy, and is one of the brain regions that has been best characterized in terms of changes in structure–function relationships after TBI (Dudek and Spitz, 1997; Nadler, 2003; Dudek and Sutula, 2007; Sutula and Dudek, 2007).

## ANIMAL MODELS OF PTE

Most information about cellular mechanisms of epileptogenesis has been derived from animal models. Although there is ongoing debate over which experimental paradigms make for appropriate models of PTE (Pitkänen and McIntosh, 2006; Dichter, 2009), the presence of spontaneous burst discharges, preferably in the form of spontaneous seizures *in vivo*, that develop following a sudden, mechanical injury (i.e., TBI) seems to be necessary minimal criteria for a “post-traumatic epilepsy” model. The presence of relatively high numbers of animals in which the behavioral, anatomical, and physiological characteristics of epileptic pathology in humans are reproduced, including short but distinct latencies between initial injury and spontaneous seizure onset, also has obvious advantages. Ideally, a model will effectively reproduce the range of tissue deformation and damage observed in mild, moderate, and severe TBI in a controllable way so that the dual pathology of cellular and molecular factors associated with injury dynamics and epileptogenesis might be dissociated. Using these selection criteria, many traditional experimental epilepsy models, such as pharmacologically induced status epilepticus, do not qualify as models of PTE, because they do not induce spontaneous seizures by a mechanical insult. This is not to negate these models, because they have provided valuable information about cellular mechanisms of epileptogenesis. Rather, these criteria provide a more restrictive and specific basis for differentiating between epileptogenesis mediated by a mechanically induced lesion versus brain insults by other causes (e.g., pharmacological agents, electrical stimulation, or models of neurodegenerative diseases). It is important to make this distinction, because there are currently few effective treatments for PTE (Temkin, 2009; discussed below). Therefore, it is important to understand how the epileptogenic cellular processes in experimental neurotrauma compare with human PTE and other experimental epilepsy models (e.g., TLE induced by status epilepticus).

### FLUID PERCUSSION INJURY

The fluid percussion injury (FPI) model is one of the best characterized in terms of epilepsy development after closed-head TBI (Pitkänen and McIntosh, 2006). In this model, injury is delivered through a craniotomy by rapid fluid injection, accomplished by a hammer swung on a pendulum. The fluid pulse first strikes the intact dura and then moves into the epidural space (Lifshitz, 2009). The height from which the pendulum hammer is dropped determines the pressure of the fluid pulse transmitted through a fluid-filled cylinder. This pressure can be used to estimate injury severity. Injury severities that have been investigated with regard to long-term changes in excitability can be categorized into two groups: severe injuries, with fluid pressure typically >3 atm; and moderate injuries, with impact forces of 2–2.2 atm. Craniotomies can be applied to the midline to produce more diffuse injuries or laterally to produce mixed focal and diffuse injury (Lifshitz, 2009).

Several studies have reported electrographic seizures in rats and more recently in mice after *severe* FPI (D’Ambrosio et al., 2004, 2005, 2009; Kharatishvili et al., 2006; Bolkvadze and Pitkänen, 2012). Susceptibility to pharmacologically induced seizures is also increased after more moderate injuries, but clear evidence for spontaneous seizures after *moderate* FPI has not been demonstrated (Kharatishvili et al., 2007; Echegoyen et al., 2009; Gurkoff et al., 2009). After severe lateral FPI, spontaneous seizures are present in up to 50% of rats (Kharatishvili et al., 2006) and 3% of mice (Bolkvadze and Pitkänen, 2012) by 12 months post-injury. Depth-electrode recordings have inferred that spontaneous electrographic seizures involve hippocampal structures, and these electrographic events are accompanied by obvious behavioral abnormalities defined by a widely used Racine rating scale for rodent seizures (Racine, 1979; Kharatishvili et al., 2006). However, seizure frequencies are typically quite low and time to first seizure is long in these animals, making it somewhat difficult to definitively attribute cellular mechanisms that occur following an injury to epileptogenesis.

In addition to lateral FPI, rats injured by rostral parasagittal FPI develop seizure-like epileptiform activity and seizures that are accompanied by more subtle changes in behavior (e.g., behavioral arrest), but do not generally develop tonic–clonic convulsive seizures (D’Ambrosio et al., 2005, 2004, 2009). Similar findings were also reported for injuries to parietal and occipital cortices, but fewer animals developed spontaneous epileptiform activity (Curia et al., 2011). Originally, behavioral seizures in this model were examined based on the traditionally used Racine rating scale (Racine, 1979; D’Ambrosio et al., 2004), but later studies developed a new seizure classification scale to describe the subtle behavioral abnormalities associated with electrographic activity in this model (D’Ambrosio et al., 2005, 2009). The authors reasoned that post-traumatic seizures after rostral parasagittal FPI did not fit well with the Racine scale and proposed that electrographic abnormalities with ≥2-s duration represent ictal events (D’Ambrosio et al., 2009). These “epileptiform” events were also observed in nearly 40% of sham-control rats by 21 weeks post-injury (D’Ambrosio et al., 2005). It is unclear why control rats also sometimes have epileptiform activity, but this may be due to how a seizure is defined (D’Ambrosio et al., 2009). Thus, it appears that the majority of electrographic abnormalities in rats injured by rostral parasagittal FPI represent relatively brief events that are associated with behavioral inactivity or crouching, compared to robust convulsive seizures observed after lateral FPI, but they may not have been considered “seizure” activity in other PTE studies (Kharatishvili et al., 2006, 2007; Hunt et al., 2009, 2010; Statler et al., 2009). Future studies that combine long-term EEG monitoring with electromyogram (EMG) and electro-oculogram (EOG) may be useful to better distinguish ictal activity from interictal events or benign variants of normal electrographic patterns that can morphologically reflect epileptiform activity but are not epileptic (Santoshkumar et al., 2009).

### WEIGHT DROP INJURY

The weight drop model, also referred to as an impact-acceleration injury, has been examined as a model of closed-head post-traumatic hyper-excitability (Golarai et al., 2001). Trauma is delivered to the neocortex by dropping a large blunt weight through a tube to impact the skull. Injury severity is managed by adjusting the height at which the weight is dropped (Marmarou et al., 2009). This injury produces large and extensive damage to cortical and subcortical structures, including the dentate gyrus and hippocampus in rats (Golarai et al., 2001). Seizures have not been reported in this model. However, increased seizure susceptibility to pentylenetetrazol (PTZ) is observed 15 weeks after injury (Golarai et al., 2001). The lack of demonstrated spontaneous seizures after weight drop is an obvious limiting factor in using this injury to model PTE. Impact is delivered to the intact skull, not directly to the brain via craniotomy as in FPI. This is sometimes considered a limitation of the model, due to increased risk for skull fracture; and injury dynamics after weight drop can depend somewhat on skull thickness (Marmarou et al., 2009). On the other hand, this characteristic might better reflect the range of variability expected in human closed-head TBI, which is unlikely to occur by craniotomy. However, by using gravitational forces to produce head injury, there can also be a risk for secondary “rebound” injury. Weight drop injury can also be difficult to perform on mice, limiting the use of transgenic animals and genetic manipulation with this model (Marmarou et al., 2009).

### CONTROLLED CORTICAL IMPACT INJURY

Controlled cortical impact (CCI) injury is a widely used experimental model of closed-head injury that was recently identified as a model of injury-induced epilepsy (Hunt et al., 2009). First developed by Lighthall (1988), this model often utilizes an electronically controlled pneumatic impactor to apply a focal contusion injury to the brain surface through a craniotomy (Dixon et al., 1991; Scheff et al., 1997; Hall et al., 2005, 2008; Saatman et al., 2006; Dixon and Kline, 2009). Injury severity is primarily managed by adjusting the depth of tissue compression, but other external injury parameters can also be controlled with precision (i.e., impact depth and velocity, impactor shape and size, and number of craniotomies). This model has unique advantages over weight drop and FPI, because it allows for good control over biomechanical parameters. This allows for a relatively consistent and reproducible focal injury with minimized risk for inaccuracy or secondary “rebound” injury. In addition, the CCI device can be scaled for use in rodents or even large animals, such as sheep or non-human primates.

Several laboratories have independently demonstrated the presence of spontaneous seizures in mice or rats after CCI. Early seizures, within 24 h of injury, have been reported in rats (Nilsson et al., 2004; Kochanek et al., 2006) and mice (Hunt et al., 2009). Within weeks after CCI, spontaneous convulsive seizures are present in up to 40% of mice after severe injuries (Hunt et al., 2009, 2010) and 9–20% of mice after more moderate injuries (Hunt et al., 2009, 2010; Bolkvadze and Pitkänen, 2012). CCI injury has also been administered to immature rats, and a percentage of these animals develop spontaneous electrographic seizures (Statler et al., 2009). Seizures after CCI are similar to spontaneous behavioral and electrographic seizures that have been described in rats after lateral FPI (Kharatishvili et al., 2006) and in models of TLE (Racine, 1979; Cronin and Dudek, 1988; Sloviter, 1992; Buckmaster and Dudek, 1997a; Patrylo and Dudek, 1998; Williams et al., 2009; Wuarin and Dudek, 2001; Shibley and Smith, 2002). Although seizure frequency after CCI injury appears considerably lower than in widely utilized chemoconvulsant TLE models, spontaneous seizures after CCI injury occur with roughly similar onset latency as pharmacologically induced TLE (Williams et al., 2009 ; Shibley and Smith, 2002), which appears to be considerably shorter than the seizure onset latency after severe lateral FPI in rats (Kharatishvili et al., 2006). For example, previous studies have suggested that limbic involvement typically does not evolve in rats until several months after FPI (D’Ambrosio et al., 2004, 2005; Kharatishvili et al., 2006), but many mice after CCI injury develop seizures and hippocampal pathology by 8 weeks post-injury.

### NEOCORTICAL UNDERCUT

This model of penetrating TBI involves partial isolation of neocortical circuits by surgical undercut to reproduce the deafferentation and white matter damage caused by neocortical TBI (Burns, 1951; Prince and Tseng, 1993). *In vivo* electrographic epileptiform activity develops in intact regions of the neocortex within hours after injury in cats (Topolnik et al., 2003), but clear evidence for spontaneous electrographic seizures has not been demonstrated.

### BLAST INJURY

Recently, a new model of penetrating, ballistic-like TBI in rats was developed to more closely recapitulate aspects of war-time TBI (Lu et al., 2011). Injury is delivered to frontal cortex using a computer-controlled hydraulic pressure generator to rapidly inflate and deflate an elastic water balloon on a probe, which creates a cortical cavity in the brain. Injury severity can be managed by amount of inflation. In this model, up to 70% of animals develop seizures 72 h after injury, and the frequency and the duration of epileptic events can be scaled according to injury severity. However, it is unknown whether rats injured by ballistic-like TBI develop long-term spontaneous seizures.

## BASIC CELLULAR MECHANISMS OF NEUROTRAUMA

**FIGURE 1:**
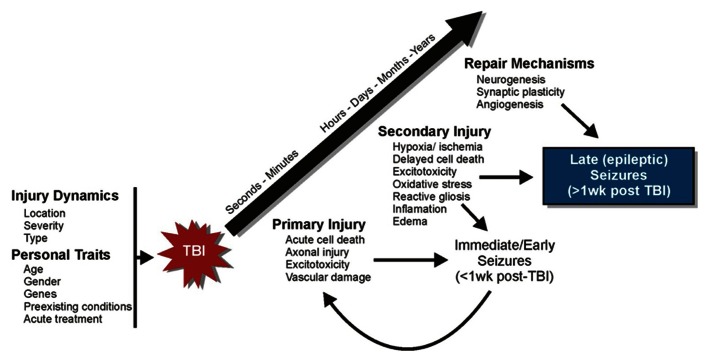
**Progression of cellular events following TBI**. A series of cellular and molecular events occur following TBI that involve three temporally overlapping responses in the brain: primary and secondary injuries and “self-repair” mechanisms.

The term “epileptogenesis” refers to a transformation process by which the normal brain develops an increased propensity for generating spontaneous seizures (Lothman et al., 1991). This process typically involves structural alterations in neural circuitry – due to progressive neuronal damage and “self-repair” mechanisms – which develop through a latent period of variable time and culminate with the emergence of spontaneous, recurrent, seizures (Dudek and Spitz, 1997). That this process includes a latent period of variable time suggests a progressive series of cellular changes may be involved. As such, human post-traumatic seizures are often classified according to the time of their presentation after injury: immediate or impact-associated (<24 h after injury), early (<1 week after injury), and late (>1 week after injury; Haltiner et al., 1997; Frey, 2003; Agrawal et al., 2006). This classification scheme is thought to represent different pathophysiological processes (Semah et al., 1998; Agrawal et al., 2006). Understanding the epileptogenic process after TBI should help to elucidate the importance of these cellular mechanisms in PTE and promote new therapeutic targets. Trauma sets into motion a multidimensional cascade of cellular and molecular events that involve three temporally overlapping responses in the brain: primary and secondary injuries and “self-repair” mechanisms (Mendelow and Crawford, 1997; Laurer et al., 2000; Graham et al., 2006; **Figure 1**). An important goal of studies which investigate post-traumatic epileptogenesis is to dissociate injury-induced cellular alterations that promote seizure generation from compensatory and “self-repair” responses.

Primary injury refers to the immediate tissue deformation and compression that occurs within seconds to minutes after mechanical brain insult (Mendelow and Crawford, 1997; Laurer et al., 2000; Graham et al., 2006). There is an immediate release of neurotransmitters, glutamate in particular, which is followed by ion channel activation and calcium influx. This can lead to excitotoxic injury, reflected by mitochondrial damage and energy depletion, neuronal and glial swelling, and cell death. Vascular damage and blood–brain barrier (BBB) disruption can also occur in primary injury. Cortical structures such as the hippocampus are especially vulnerable to neuronal damage after moderate and severe TBI. Moreover, the presence of immediate or early seizures can further exacerbate initial damage and complicate injury management (Temkin, 2009). These seizures are considered to be injury-induced (i.e., provoked) and are not epileptic, because they occur as a direct result of neurologic and systemic abnormalities of the acute trauma. Strategies to protect against primary injury are typically aimed at taking preventive rather than therapeutic measures (e.g., wearing protective headgear).

Secondary injury involves a myriad of cellular and physiological factors associated with progressive tissue damage (Mendelow and Crawford, 1997; Teasdale and Bannan, 1997; Laurer et al., 2000; Graham et al., 2006). Over time, brain injury triggers inflammatory cascades, growth factor responses, edema, mitochondrial dysfunction, oxidative stress due to the build-up of free radicals and reactive oxygen species, delayed cell death, perturbation of cellular calcium homeostasis, and hypoxia and ischemia. The brain also initiates “self-repair” mechanisms over the course of days to months concurrent with the development of secondary tissue damage. This period is characterized by synaptic circuit remodeling, axon sprouting, synaptic plasticity, gliosis, neurogenesis, and angiogenesis. The time-dependant reorganization of synaptic circuitry can promote increased synchronous neuronal activity that contributes to functional recovery, but may also contribute to spontaneous seizure generation. During this period, seizures that occur spontaneously (i.e., late seizures) are considered to represent PTE because they reflect permanent changes in neuronal structure and function. The primary goal of many neuroprotective measures is to prevent or reduce secondary brain damage and to enhance beneficial “self-repair” mechanisms (Mendelow and Crawford, 1997; Teasdale and Bannan, 1997; Graham et al., 2006). However, repair mechanisms may not be universally beneficial, as some of these same mechanisms (e.g., axon sprouting and synaptic reorganization) are also correlated with the development of spontaneous seizures.

Genetics, gender, age, acute medical treatments, and the agent of injury all likely influence the epileptogenic processes, but the contribution of these personal traits and injury dynamics in epilepsy have not been well established (Pitkänen and McIntosh, 2006). A better understanding of the importance of these factors in post-traumatic epileptogenesis will likely elucidate why some individuals develop PTE after TBI while others do not.

## NEURAL CIRCUIT REORGANIZATION

The central dogma in epilepsy research has long been that seizures occur due to some type of imbalance between excitatory and inhibitory neurotransmission (Dudek and Spitz, 1997; McCormick and Contreras, 2001; Nadler, 2003). In humans and animal models of injury-induced epilepsy, the presence of spontaneous seizures is strongly associated with axon sprouting and reorganization of neural circuitry. Within this framework, most experimental injury models show evidence that excitatory connectivity is enhanced and inhibitory influences are decreased. These changes often involve the presence of recurrent excitatory circuits, which form when principal cells are sufficiently interconnected and have long been proposed as a cellular basis for pathologically synchronous neural activity and seizures (Traub and Wong, 1982; Dudek and Spitz, 1997; McCormick and Contreras, 2001).

### EXCITATORY CIRCUIT CHANGES

**FIGURE 2:**
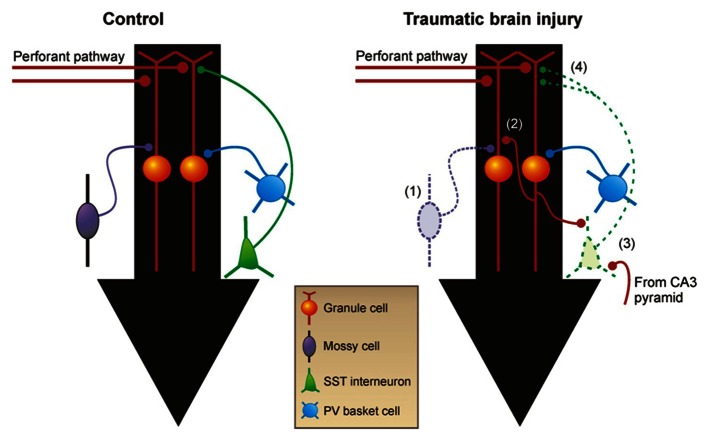
**Reorganization of neural circuitry in the dentate gyrus after brain injury**. (1) Within days after injury, there are fewer excitatory mossy cells and inhibitory GABA neurons in the hilus. (2) Within weeks after TBI, mossy fibers sprout axon collaterals into the inner molecular layer and form new recurrent excitatory circuits among granule cells. (3) Somatostatin-positive GABA neurons in the hilus that survive TBI receive increased excitatory input from granule cells and CA3 pyramidal cells. (4) These neurons also have increased axon length in the molecular layer in acquired epilepsy models. Black arrows show direction of information flow through the dentate gyrus. PV, parvalbumin; SST, somatostatin.

Several laboratories have independently demonstrated reactive plasticity of glutamatergic axons and formation of new, recurrent excitatory circuits in TBI models (Santhakumar et al., 2000; Kharatishvili et al., 2006, 2007; Hunt et al., 2009, 2012). The dentate gyrus has acted as a model system to study excitatory axon sprouting associated with PTE because it is particularly susceptible to injury and subsequently undergoes structural reorganization (**Figure 2**). Dentate granule cells, which are not normally connected to each other, sprout axon collaterals into the inner molecular layer to form functional recurrent excitatory connections with nearby granule cells during epileptogenesis (Cronin and Dudek, 1988; Cronin et al., 1992; Wuarin and Dudek, 1996, 2001; Lynch and Sutula, 2000; Winokur et al., 2004), a process termed “mossy fiber sprouting.” These dramatic changes in local mossy fiber circuits are relatively easy to detect by Timm’s histochemistry and are consistently reproduced in human tissue (de Lanerolle et al., 1989; Sutula et al., 1989; Houser et al., 1990; Babb et al., 1991; Zhang and Houser, 1999) and experimental models of TLE (Nadler et al., 1980; Ben-Ari, 1985; Tauck and Nadler, 1985; Cronin and Dudek, 1988; Buckmaster and Dudek, 1997b; Buckmaster et al., 2002; Shibley and Smith, 2002). Mossy fiber sprouting into the inner molecular layer of the dentate gyrus is also a common feature of the epileptic dentate gyrus in TLE patients with a history of head injury (Swartz et al., 2006), and it has been consistently reported in animal models of TBI (Santhakumar et al., 2000; Kharatishvili et al., 2006, 2007; Hunt et al., 2009, 2012). Post-traumatic mossy fiber sprouting is generally more robust after severe versus mild TBI and might be related to whether cortical injury directly impinges upon the hippocampus (Hunt et al., 2012). However, the degree of mossy fiber sprouting after experimental TBI is qualitatively less than the robust, bilateral sprouting observed after experimental status epilepticus, and it is often more regionally localized to areas near the injury.

These anatomical changes in mossy fiber circuits are associated with a functional alteration in neuronal circuitry of dentate granule cells that may contribute to abnormal network synchronization after TBI. Similar to rodent models of TLE (Cronin et al., 1992; Wuarin and Dudek, 1996, 2001; Patrylo and Dudek, 1998; Lynch and Sutula, 2000; Winokur et al., 2004), new functional recurrent excitatory circuits emerge in the presence of mossy fiber sprouting after TBI that are not detected in the normal dentate gyrus (Hunt et al., 2009, 2010). When surgically isolated from afferent input (i.e., entorhinal cortex), spontaneous and evoked reverberating burst discharges, indicative of synchronous network activation, have been observed by single-cell and extracellular field potential recordings from granule cells in slices with mossy fiber sprouting after TBI (Hunt et al., 2009, 2010). Studies using localized glutamate stimulation have suggested monosynaptic granule cell-to-granule cell connections in slices with mossy fiber sprouting, which are absent in the normal dentate gyrus (Hunt et al., 2010). These new excitatory connections among granule cells are only present in brain slices with mossy fiber sprouting and are not detected in slices without sprouting, even in the same injured animal. Although recurrent excitatory circuits are normally masked by recurrent inhibitory circuits and can only be revealed by experimentally altering the extracellular environment to increase excitation, these new excitatory circuits have been proposed to form the basis from which synchronous network activity can periodically arise in the dentate gyrus, particularly if inhibition periodically fails. Therefore, mossy fiber sprouting may provide a means for regional granule cell network synchronization after TBI that may be “unmasked” if inhibitory control is impaired (Patrylo and Dudek, 1998).

Other brain regions that are susceptible to injury, such as hippocampus and neocortex also undergo synaptic reorganization after TBI. For example, an increase in spontaneous and evoked burst discharges has been detected in neocortex of rats within 2 weeks after severe CCI injury (Yang et al., 2010). Although it is not known whether these changes are due to the formation of new excitatory circuits in neocortex or enhanced intrinsic excitability of existing circuits after injury, similar findings have been observed at similar time points after neocortical undercut (recently reviewed by Prince et al., 2012), which appear to be associated with an increase in excitatory synapses onto pyramidal neurons. In CA1 of hippocampus, pyramidal neurons show immediate deafferentation following CCI injury, possibly due to loss of CA3 neurons, which is followed by a time-dependent increase in number of synaptic contacts (Scheff et al., 2005) and fiber excitability (Norris and Scheff, 2009). Taken together, these findings suggest that changes in functional excitability of other brain regions might also contribute to seizure generation after TBI, independent of mossy fiber sprouting.

### INHIBITORY CIRCUIT CHANGES

**FIGURE 3:**
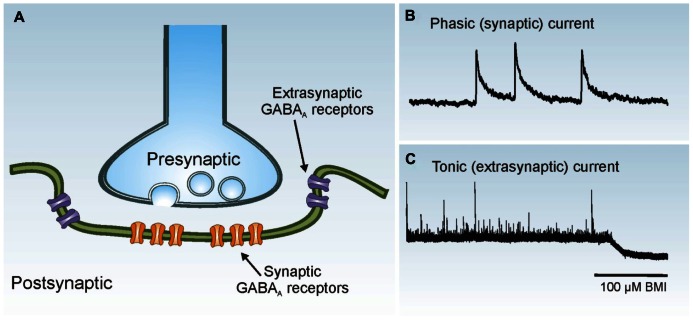
**Phasic and tonic GABA_**A**_ receptor activation**. **(A)** Synaptic receptors (orange) are located on the postsynaptic membrane just beneath presynaptic release sites, whereas extrasynaptic receptors (purple) are located away from the synaptic junction. **(B)** Phasic (synaptic) IPSCs are rapid events. Three individual IPSCs are shown in a whole-cell voltage-clamp recording obtained from a dentate granule cell. **(C)** The tonic (extrasynaptic) current in this granule cell is revealed as a baseline shift after application of the GABA_A_ receptor antagonist bicuculline methiodide (BMI; 100 μM).

GABAergic interneurons form robust local synaptic connections with principal cells and play an important role in controlling cortical activity. Two primary modes of GABA_A_ receptor-mediated inhibition have been identified: phasic (synaptic) and tonic (extrasynaptic) inhibition (Farrant and Nusser, 2005; Glykys and Mody, 2007; **Figure 3**). Phasic inhibition refers to the transient activation of postsynaptic GABA_A_ receptors at the synaptic junction following exposure to high concentrations of GABA released from presynaptic vesicles. This form of synaptic communication allows for rapid transmission of information from the presynaptic terminal to the postsynaptic membrane in a spatially and temporally restricted manner. In whole-cell voltage-clamp recordings, synaptic events can be easily identified as inhibitory postsynaptic currents (IPSCs). Small amounts of GABA escape the synaptic cleft and activate high-affinity extrasynaptic GABA_A_ receptors on the same or adjacent neurons, an event termed “spillover.” Tonic inhibition refers to the persistent activation of these extrasynaptic GABA_A_ receptors by low concentrations of ambient GABA. Phasic and tonic currents can be isolated by high concentrations of GABA_A_ receptor antagonists. In whole-cell voltage-clamp recordings the tonic current is reflected by a shift in “holding” current.

In the dentate gyrus, the loss of certain populations of hilar interneurons is a common histopathological feature in human PTE (Swartz et al., 2006), and several labs have reported a loss in the number of inhibitory interneurons after experimental TBI (Lowenstein et al., 1992; Toth et al., 1997; Santhakumar et al., 2000; Grady et al., 2003; Hall et al., 2005). Although it is unclear whether subtypes of hilar neurons are preferentially lost after TBI, as is often observed in TLE, inhibitory hilar neuron loss ipsilateral to the site of injury is typically associated with a reduction in synaptic inhibition of granule cells (Hunt et al., 2011; Pavlov et al., 2011; Gupta et al., 2012). In contrast, whether there are changes in tonic inhibition after TBI remain unresolved. One study reported an increase in tonic current amplitude in dentate granule cells contralateral to severe CCI injury (Mtchedlishvili et al., 2010), whereas other studies reported no change in tonic current amplitude to granule cells (Pavlov et al., 2011) or a marked decrease in tonic inhibition in semilunar granule cells (Gupta et al., 2012) ipsilateral to moderate FPI. Recent evidence has also shown that there are changes in the expression of GABA_A_ receptor subunit levels after CCI injury (Mtchedlishvili et al., 2010; Gupta et al., 2012; Raible et al., 2012), which might also affect inhibitory influences after TBI.

Beyond the presence of substantial interneuron loss and a subsequent reduction in synaptic inhibition of granule cells after TBI, relatively little is known about how individual interneuron subpopulations are affected by mechanical trauma. In a recent study, we reported an increase in excitatory input to hilar somatostatin interneurons arising from granule cells and CA3 pyramidal neurons after CCI injury, suggesting that reactive plasticity of glutamatergic axons is not limited to connections among principal neurons (Hunt et al., 2011). These changes were accompanied by an increase in the decay time constant of spontaneous inhibitory synaptic events in granule cells, which is consistent with increased activity or axon sprouting of this dendritically projecting GABA neuron population after injury. Similar changes have also been reported in a model of status epilepticus (Zhang et al., 2009; Halabisky et al., 2010). However, whether this phenomenon represents a compensatory response to boost feedback inhibition in the dentate gyrus, which is compromised by injury, or a pathological response that contributes to seizures (e.g., by a periodic failure of already fragile inhibitory circuits or increased risk for GABA-mediated depolarization in distal dendrites) is unknown. It is also not known how other populations of inhibitory neurons are affected by TBI. Given the relatively large number of ways in which inhibitory circuits can be modified by head injury and the important role of inhibitory neurons in restraining excessive cortical excitability (Chagnac-Amitai and Connors, 1989; Trevelyan et al., 2007; Schevon et al., 2012), future studies aimed at understanding how inhibition is modified after closed-head TBI, including in brain regions outside of the dentate gyrus, should remain an active area of investigation.

## BRAIN INFLAMMATION AND BLOOD–BRAIN BARRIER DYSFUNCTION AFTER TBI

While the immune system provides crucial protection against infected or damaged tissue, accumulating evidence points to an involvement of inflammation in the pathophysiology of epilepsy. Heightened or abnormal innate immune responses are noted in individuals with epilepsy, in animal models of this disease, as well as with pro-epileptic insults such as infection, trauma, ischemia/hypoxia, fever, and recurrent seizures (Nelson and Ellenberg, 1976; Pacifici et al., 1995; Carpio et al., 1998; Crespel et al., 2002; Kotila and Waltimo, 2005; Choi and Koh, 2008; Vezzani et al., 2013; Dedeurwaerdere et al., 2012). Importantly, many innate immune processes play significant roles in cell excitability and survival during inflammation, which have the potential to promote network hyper-excitability (Hama et al., 1991; Yamada and Hatanaka, 1994; Schäfers and Sorkin, 2008; Vezzani et al., 2008). Experimental models of epilepsy that do not involve head injury (non-PTE models) have described several inflammatory pathways that contribute to seizure susceptibility (Dedeurwaerdere et al., 2012). Despite these indications, surprisingly very few basic studies have studied the role of the immune system in experimental PTE even though inflammation and BBB breakdown are hallmark features of head injury (Schmidt et al., 2005; Werner and Engelhard, 2007). The causal role of inflammation in PTE development can, at least in part, be assessed by systematic experimental manipulation of individual components of inflammation in animal models. An important consideration in these studies is to discriminate between acute effects of seizure threshold (i.e., anti-convulsant effects) and long-term development of spontaneous seizures (i.e., epileptogenesis), because these processes may have different mechanisms and inflammation may contribute to them in different ways. This section attempts to highlight the need to examine the role of inflammation in epileptogenesis as presently there a very few studies that do so using PTE or non-PTE models.

Work in brain trauma has extensively characterized patterns of immune responses following TBI (detailed review in Morganti-Kossmann et al., 2001; Lucas et al., 2006). Head trauma initially triggers inflammation by a variety of factors including direct damage to the BBB as well as an accumulation of foreign particles, extravasated blood proteins, cellular debris, complement fragments, prostaglandins, and both reactive oxygen and nitrogen species within the brain (Dardiotis et al., 2012). The resulting inflammatory cascade involves the coordinated activity of resident central nervous system (CNS) immune and non-immune cells, circulating immune cells, and transport mechanisms across the BBB. Neurons, astrocytes, and particularly microglia located proximal to the site of injury rapidly respond to trauma with pro-inflammatory signals including chemokines, cytokines, and tissue adhesion molecules (Ransohoff, 2002). The release of these inflammatory mediators supports the further recruitment, migration, and infiltration of leukocytes into the brain parenchyma. The invasion of leukocytes (chiefly neutrophils, monocytes, and lymphocytes) results in further disruption of homeostasis and secretion of pro-inflammatory signals within acutely spared brain regions resulting in a potent inflammatory response to these sites (Morganti-Kossmann et al., 2001). Additional increases in BBB permeability magnify levels of inflammatory units and blood proteins entering brain tissue (Dardiotis et al., 2012).

In the context of epilepsy, an important feature of inflammation is its contribution to ongoing cell death after trauma. In addition to cell loss by excitotoxicity, ischemia, and disruptions in fluid and metabolic homeostasis, inflammatory cytokines enhance signaling components of apoptosis (Morganti-Kossmann et al., 2001). Proteases and free radicals released during inflammation support lipid and protein peroxidation, mitochondrial damage, DNA damage, as well as further induction of apoptotic mechanisms (Tyurin et al., 2000). Conversely, many immune components provide a neuroprotective effect following trauma. The mechanisms underlying these neuroprotective effects are not fully characterized, however inflammatory components can provide neurotrophic support by increasing growth factors, reducing oxidative stress, or via anti-inflammatory signaling (Morganti-Kossmann et al., 2001). Understanding the role that individual inflammatory components serve in post-traumatic cell loss will likely help determine how they affect PTE development. Components of inflammation also affect cell excitability within the CNS in addition to their effects on cell survival. For example, inflammatory cytokines modulate neurotransmitter levels (Zalcman et al., 1994; Dunn, 2006), communication between neurons and glia (Hanisch, 2002), GABA_A_ receptor-mediated responses (Miller et al., 1991), and calcium currents (Plata-Salamán and Ffrench-Mullen, 1992; Qiu et al., 1998). Chemokines also affect neuronal excitability by modulation of voltage-dependent channels (sodium, potassium, and calcium), activation of an inward rectifying potassium conductance and increasing the release of neurotransmitters including GABA, glutamate, and dopamine (Fabene et al., 2010). The effects of inflammation on cell loss and cell excitability may serve a role in the development of network hyper-excitability and epileptogenesis following TBI.

There are several challenges to understanding the overall contribution of inflammatory pathways to PTE. Individual components of inflammation typically affect many processes; they are pleiotropic, and are integrative such that one process can affect many others via forward and backward regulatory pathways. For example, treatment with antibodies against interleukin (IL)-6 results in elevated serum levels of tumor necrosis factor alpha (TNF-α) whereas treatment with antibodies against TNF-α results in decreased serum levels of IL-6 (Starnes et al., 1990). Individual inflammatory components also have different temporal expression patterns following head injury (for review, see Korn et al., 2005; Lucas et al., 2006). There is also a complex spatial patterning of post-traumatic inflammation where expression may be specific to cell-type or brain region and may be different between peripheral and central tissues (Morganti-Kossmann et al., 2001; Schmidt et al., 2005; Ravizza and Vezzani, 2006). For example, transgenic mice that over-express IL-6 under a glial fibrillary acidic protein (GFAP) promoter (i.e., to reflect astrocytic production of IL-6) exhibit spontaneous seizures, neuronal loss, neural dysfunction, and breakdown of the BBB (Campbell et al., 1993). In contrast, mice that over-express this same cytokine under a neuron-specific enolase (NSE) promoter display no neuronal damage or seizures (Fattori et al., 1995). All of these characteristics depend on the type of immune component, unique biomechanics of the TBI, as well as the specific traits of the injured individual (Rovegno et al., 2011). As studies continue to document these pathways in TBI it is becoming increasing important to ascertain more specific profiles of individual inflammatory components that include their spatial and temporal patterning as well as their net effect on cell loss and cell excitability.

The PTE field can be guided by reports that characterize or manipulate immune processes in non-PTE models and models of trauma. To date, many links between innate immunity and seizure expression in non-PTE models have been made studying cytokine signaling (Dedeurwaerdere et al., 2012). Cytokines are a diverse family of glycoproteins that are secreted by glia, neurons, leukocytes, endothelial and epithelial cells in response to stress, immune challenge, and injury (Ransohoff, 2002). Cytokine signaling occurs at receptors found on a variety of cells throughout the body via transcription-dependent and transcription-independent mechanisms (Bezbradica and Medzhitov, 2009). Cytokines are often characterized as either pro-inflammatory (e.g., IL-1β, IL-6, and TNF-α) or anti-inflammatory, with the latter exerting their effects by reducing expression of pro-inflammatory cytokines (e.g., IL-4, IL-10, and IL-13; Kadhim et al., 2008). While basal cytokine expression is low, these signals are highly elevated in the acute phase of TBI (Ransohoff, 2002). Transgenic glial over-expression of IL-6 (Campbell et al., 1993) or TNF-α (Akassoglou et al., 1997) results in spontaneous seizures and neurodegeneration including a loss of GABAergic cells in cortical and hippocampal structures. IL-6 delivered to naïve animals intranasally increases their seizure susceptibility to the convulsant PTZ (Kalueff et al., 2004). Brain administration of IL-1β increases the duration of seizures induced acutely by kainic acid, whereas administration of the IL-1 receptor antagonist (IL-1ra) provides anti-convulsant effects (Vezzani et al., 2000). Mice with transgenic glial over-expression of IL-1ra or IL-1 receptor type 1 knock-out mice exhibit reduced susceptibility to convulsant-induced seizures (Vezzani et al., 2000, 2002). The prostaglandin receptor EP2 has recently been targeted using novel inhibitors because of its role in diverse cytokine signaling and neurotoxicity (Jiang et al., 2012). Inhibition of prostaglandin receptor EP2 reduces cytokine expression, markers of astrogliosis and microgliosis, mortality, hippocampal neurodegeneration, and BBB leakage in pilocarpine-treated mice in the absence of acute anticonvulsant effects (Jiang et al., 2012, 2013). These results, particularly the long-term development of spontaneous seizures observed with over-expression of IL-6 and TNF-α indicate a role for cytokines in epileptogenesis that needs to be assessed in models of PTE.

Increases in BBB permeability and tissue adhesion molecules may also reduce seizure threshold or have pro-epileptogenic effects (Quadbeck and Helmchen, 1958; Kasantikul et al., 1983; reviewed in Shlosberg et al., 2010; Kim et al., 2012). The BBB’s constituent components: endothelial cells, pericytes, and astrocytic end-feet work in concert to homeostatically regulate the flow of molecules and cells between the vasculature and brain parenchyma (Wolburg and Lippoldt, 2002). The resulting “neurovascular unit” that arises from these structures in combination with local neurons is drastically altered by primary and secondary damage following TBI (Korn et al., 2005). While a positive correlation between BBB permeability and recurrent seizures has been observed following electrically induced status epilepticus (van Vliet et al., 2007), the relationship between seizures and BBB permeability remains complex (Cornford and Oldendorf, 1986; Janigro, 1999; Tomkins et al., 2001; Pavlovsky et al., 2005). Increased BBB permeability allows entry of inflammatory cells/molecules into the brain, while in parallel, inflammatory signaling from sites within the brain can also promote BBB permeability (Abbott, 2000; Huber et al., 2001; Marchi et al., 2007; Zlokovic, 2008). Thus, secondary phases of increased BBB permeability and inflammation can occur in response to immune signaling and these secondary phases may play a role in post-traumatic epileptogenesis (Korn et al., 2005).

Emerging work using animal models has further characterized how BBB permeability is altered in pathological conditions and how individual infiltrates within the extracellular space affect local cell survival and excitability (Seiffert et al., 2004; Ivens et al., 2007; Fabene et al., 2008; Tomkins et al., 2008). Repeated administrations of a granulocyte-specific antibody to reduce populations of neutrophils produce anti-convulsant and anti-epileptogenic effects in pilocarpine mice (Fabene et al., 2008). Brain slices from animals given experimental disruption of the BBB using bile salts exhibit epileptiform activity (Seiffert et al., 2004). These bile salts result in an extravasation of serum albumin into the brain and this epileptiform activity is similarly observed in slices from animals given direct cortical application of serum albumin (Seiffert et al., 2004). The mechanistic link between extravasation of serum albumin into the brain and epileptiform activity is suggested to be a transforming growth factor beta (TGF-β)-dependent down-regulation of an inwardly rectifying K^+^ current in astrocytes that reduces their ability to buffer extracellular K^+^ (Ivens et al., 2007; Cacheaux et al., 2009; David et al., 2009). Administration of rapamycin (van Vliet et al., 2012) or an antagonist of the prostaglandin receptor EP2 (Jiang et al., 2013) are both associated with reduced BBB leakage. Administration of IL-1β or transgenic over-expression of TNF-α result in increased BBB permeability (Akassoglou et al., 1997; Ferrari et al., 2004; Ravizza et al., 2008). There is also growing interest in understanding how adhesion molecules that support transport across the BBB may contribute to, as opposed to result from, epileptic pathologies (Dedeurwaerdere et al., 2012). Pilocarpine-induced seizures increase levels of vascular cell adhesion molecules (VCAMs) including intercellular adhesion molecule-1 (ICAM-1), VCAM-1 and P-selectin glycoprotein ligand-1 (PSGL-1) and these increases enhance leukocyte adhesion to CNS vessels *in vivo* (Fabene et al., 2008). Exogenous delivery of antibodies for either α4 integrin or VCAM-1 delivered 1 h after pilocarpine treatment, and repeated (every other day for 20 days), greatly reduces spontaneous seizure expression (Fabene et al., 2008). Interestingly, magnetic resonance imaging of individuals with TBI identifies increased BBB permeability in the majority of cases involving PTE and this enhanced permeability is sustained from days to years post-injury in some individuals (Tomkins et al., 2008). These findings suggest that BBB permeability may be a common feature of inflammation-dependent changes in seizure threshold and/or epileptogenesis. The long-term suppression of spontaneous seizures in pilocarpine-treated mice by *in vivo* treatment with antibodies against α4 integrin, VCAM-1, or granulocytes indicates several important potential targets to treat PTE.

Chemokines (also known as chemoattractant cytokines) may also contribute to the development of epilepsy. Chemokines are chemotactic proteins that are characterized by the relative location of their cysteine (Cys) residues within the NH_2_ terminal of each protein. Chemokines are classified as CC, CXC, CX_3_C, and XC where CC refers to both Cys residues adjacent to one another, CXC refers to both Cys residues separated by one amino acid, CX_3_C refers to both Cys residues separated by three amino acids and XC has only one Cys residue (Moser et al., 2004). There is also a lipopolysaccharide inducible CC chemokine receptor termed L-CCR (Shimada et al., 1998). The central role of chemokines is to direct cell migration including migration of leukocytes during brain inflammation (Ley et al., 2007). Importantly, individual (or classes) of chemokines are known to affect specific types of leukocytes (Moser et al., 2004). Many chemokines are increased after head trauma including CCL3, CCL9, CCL12, CCL10, and CCL2 (also known as monocyte chemoattractant protein MCP-1; Israelsson et al., 2008). Unfortunately few, if any, studies have selectively targeted chemokines to characterize their role in PTE. CCL2 is up-regulated following pilocarpine or kainate induced SE (Fabene et al., 2010). CCL3 and CCL4 are increased in amygdala stimulation induced SE (Guzik-Kornacka et al., 2011). Administration of the small molecule Minozac results in reductions in CCL2, IL-1β, IL-6, and TNF-α; behavioral improvement; and attenuates the increased susceptibility to electroconvulsive shock-induced seizures after experimental closed-head injury (Lloyd et al., 2008; Chraszcz et al., 2010). These results indicate a potential neuroprotective or neurorestorative role for suppressors of chemokines, cytokines, or other pro-inflammatory responses (Lloyd et al., 2008). Targeted approaches are needed to assess the contribution of individual chemokines to epileptogenesis in models of PTE. Identification of a role for individual chemokines can provide clues as to what types of infiltrating leukocytes are pro-epileptogenic based on established relationships between individual chemokines and their target immune cells.

Collectively, a number of studies suggest that inflammation may contribute to the development of PTE. Inflammation or other immune changes are shared features of many seizure disorders and these changes often precede the manifestation of behavioral seizures (i.e., during the latent period; Majores et al., 2004; Ravizza et al., 2008). An emerging concept is that seizures themselves can result in brain inflammation, thereby raising the possibility of a reciprocal relationship between inflammation and seizures that may support or maintain epilepsy (Librizzi et al., 2012). Inflammation affects cell excitability and cell survival and there is some evidence that these effects can promote network hyper-excitability (Miller et al., 1991; Plata-Salamán and Ffrench-Mullen, 1992; Zalcman et al., 1994; Qiu et al., 1998; Ravizza and Vezzani, 2006). There is also a growing need to distinguish immune effects on seizure mechanisms from immune effects on recovery and compensation as there is at least some evidence that individual inflammatory components affect both processes (Morganti-Kossmann et al., 2001). For example, in addition to TNF-α’s pro-excitatory and pro-epileptic effects, this inflammatory cytokine serves a neuroprotective role, since mice lacking TNF-α show enhanced lesion size and BBB breakdown following CCI (Sullivan et al., 1999). The pleiotropic and integrative nature of inflammatory components will offer some difficulties in this type of pathway-specific targeting. Nonetheless, this pathway-specific approach needs to be further extended in models of PTE and other forms of epilepsy to characterize how individual components of inflammation affect epileptogenesis. While there are clear challenges in identifying the role of inflammatory processes in the pathophysiology of PTE, the possibility to reduce or block the development of epilepsy after TBI provides great encouragement for work in this area.

## SUMMARY

The development of medically intractable epilepsy is one of the most common long-term health problems associated with head injury in humans. Recent work using animal models of TBI has shown substantial evidence for the formation of new, excessive recurrent excitatory synaptic connectivity and alterations to inhibitory interneurons in this disorder, although these studies have primarily focused on the dentate gyrus and other regions of the injured brain should be investigated in more detail. However, there are a number of important aspects of post-traumatic epileptogenesis that are not fully understood. For example, there is a need for a more complete profile of how individual components of inflammation and BBB permeability contribute to post-traumatic epileptogenesis. Thus, much progress has been made in modeling epilepsy in rodents after mechanical injury and basic mechanisms of neural circuit reorganization associated with epilepsy have been identified in these models, but new developments in this field should continue to identify precise cellular or molecular mechanisms that control the development of epilepsy after head injury.

## Conflict of Interest Statement

The authors declare that the research was conducted in the absence of any commercial or financial relationships that could be construed as a potential conflict of interest.
